# The effect and safety of dexmedetomidine as an adjuvant to local anesthetics in erector spinae plane block: a systematic review and meta-analysis of randomized controlled trials

**DOI:** 10.1186/s12871-023-02019-x

**Published:** 2023-02-27

**Authors:** Liang Yu, Xiaojuan Shen, He Liu

**Affiliations:** 1 Department of Anesthesiology & Huzhou Key Laboratory of Basic Research and Clinical Translation for Neuromodulation, Huzhou Central Hospital (The Affiliated Huzhou Hospital, Zhejiang University School of Medicine; The Fifth School of Clinical Medicine of Zhejiang Chinese Medical University; Affiliated Central Hospital Huzhou University), No. 1558, Sanhuan North Road, Wuxing District, Huzhou, 313003 Zhejiang Province People’s Republic of China; 2 706A Ward, Huzhou Central Hospital (The Affiliated Huzhou Hospital, Zhejiang University School of Medicine; The Fifth School of Clinical Medicine of Zhejiang Chinese Medical University; Affiliated Central Hospital Huzhou University), No. 1558, Sanhuan North Road, Wuxing District, Huzhou, 313003 Zhejiang Province People’s Republic of China

**Keywords:** Anesthesia adjuvants, Erector spinae plane block, Dexmedetomidine, Postoperative pain

## Abstract

**Background:**

Dexmedetomidine (DEX) has been thought to be an effective adjuvant to local anesthetics (LAs) in erector spinae plane block (ESPB), however, this method of use is not recorded in the drug instructions. Hence, our meta-analysis will evaluate its efficacy and safety for the first time.

**Methods:**

A systematic search of published articles was conducted in the PubMed, Embase, Web of science, and Cochrane Library databases up to July 17, 2022, using specific keywords related to our aims. The time first to request rescue analgesia, number of patient controlled intravenous analgesia (PCIA) presses, rate of rescue analgesia use, postoperative nausea and vomiting (PONV), arrhythmia, and hypotension were calculated by using random-effect models. This systematic review and meta-analysis was registered with PROSPERO (registration number: CRD42022345488).

**Results:**

Numerous electronic databases were searched and finally 8 studies with a total of 570 patients, 303 in the DEX arm, 267 in the control arm were included. As an adjuvant to LAs, DEX significantly increased the time to first request of rescue analgesia (mean difference [MD] = 8.40, 95% confidence interval [CI] = 4.70–12.10, *P* < 0.00001), reduced the number of PCIA presses (MD = -4.12, 95% CI = -7.79 to -0.45, *P* = 0.03) and the rate of rescue analgesia (odds ratio [OR] = 0.33, 95% CI = 0.17–0.65, *P* = 0.002). Moreover, the combination reduced the risk of PONV (OR = 0.57, 95% CI = 0.36–0.91, *P* = 0.02). In addition, there was no difference in the incidence of hypotension (OR = 1.01, 95% CI = 0.37–2.74, *P* = 0.99) and arrhythmia (OR = 0.76, 95% CI = 0.19–3.07, *P* = 0.70).

**Conclusion:**

DEX can reduce analgesic requirements after various surgical procedures when used as an adjuvant to LAs for ESPB. Moreover, there was no significant difference between the two groups in terms of safety indicators (arrhythmia, hypotension).

**Supplementary Information:**

The online version contains supplementary material available at 10.1186/s12871-023-02019-x.

## Introduction

In 2016, Forero et al. reported for the first time that this technique had been successfully implemented for the treatment of thoracic neuropathic pain [1]. The new regional blocking technique can utilize to reduce postoperative pain effectively in various surgical procedures such as breast, thoracic, abdominal and lumbar surgery [2, 3]. However, although usually the use of long-acting local anesthetics (LAs), the duration of pain relief is only about 10 h [4]. Even though continuous catheter-based nerve block can prolong the postoperative pain relief time, placing them requires additional time and cost, and increases the risk of infection and neurological complications [5]. To remedy this shortcoming, various adjuvant drugs, such as opioids [6], dexamethasone [7], and buprenorphine [8], have been used in combination with LAs to prolong the duration of analgesia with varying degrees of success.

Dexmedetomidine (DEX) is a highly selective alpha-2 adrenergic receptor agonist [9]. In previous clinical studies, DEX as adjuvant to LAs has been the subject of increasing interest as the potential to prolong blockade duration [10]. Mechanisms of DEX in nerve block are as follows: DEX inhibits sodium channels and potassium currents in neurons and blocks the hyperpolarization-activated cyclic nucleotide-gated channel leading to the enhancement of activity-dependent hyperpolarization [11, 12]. More and more clinical studies have used DEX as an adjuvant to LAs for ESPB. Although some studies have been completed, only the intravenous route for DEX is approved by the U.S Food and Drug Administration (FDA). So far, the efficacy and safety of DEX combined with LAs in the ESPB have not been systematically reviewed. Therefore, the purpose of this study was to clarify its effect and safety by combining the indicators of postoperative analgesia and adverse reactions.

## Methods

This meta-analysis was planned and conducted according to the Preferred Reporting Items for Systematic Reviews and Meta-Analyses (PRISMA) checklist [13]. The authors registered the protocol in the International Prospective Register of Systematic Reviews (registration number: CRD42022345488). This study did not require ethics approval or informed consent as no patient information was collected.

### Search strategy

We searched the electronic database including PubMed, Embase, Web of science, and Cochrane Library from the establishment of the database to July 17, 2022. In addition to the above databases, the database used internally in the author's work unit was retrieved as a supplement, named “other database”. The procedure of searching was systematically performed by 2 researchers (Liang Yu and Xiaojuan Shen) independently without language restrictions. Following search terms are used: ecector spinae plane block and Dexmedetomidine. Appropriate adjustments were made when searching the database and if the full-text article was available. The search strategies for each database are summarized in supplementary material (Additional file 1).

### Inclusion and exclusion criteria

Studies were included if they met the following criteria: (1) randomized controlled trials (RCTs); (2) Surgical patients with and without DEX as an adjuvant to LAs in ESPB; (3) data regarding postoperative pain and side effect. Conversely, the following types of articles were excluded: articles other than original research (such as: review articles or commentaries); case reports; irrelevant trials; duplicate reports; conference abstract and letters. There were no restrictions on publication year, publication language, or publication region. In case of any discrepancy in the included studies between the two authors, a senior author ( He Liu) participated in the study selection and made the final decision.

### Data extraction

Two authors (Liang Yu and Xiaojuan Shen) who independently examined the final RCTs screened and collected the following data: first author, publication year, country, surgery, sample size, types of LAs, DEX dosage, block localization, primary outcome. Medians, interquartile ranges, and ranges were approximated as means and standard deviations (SDs) using the quantile estimation method and the Box-Cox method of McGrath et al. [14] as well as the method for unknown non-normal distributions approach of Cai et al. [15]. Data from trials with more than two intervention groups receiving different doses of perineural DEX were combined into a single group as per the Cochrane Handbook [16]. If two independent examiners cannot agree, a third reviewer (He Liu) made the final decision.

### Primary and secondary outcome

The time initially to request rescue analgesia was defined as primary outcomes. The secondary outcome included the number of PCIA presses, rate of rescue analgesia use, incidence of PONV, arrhythmia, and hypotension.

### Risk of bias

Two authors (Liang Yu and Xiaojuan Shen) independently evaluated the risk of bias and quality of evidence. The risk of bias was assessed using the Cochrane risk-of-bias tool for RCTs, which consists of seven sources of bias: random sequence generation, allocation concealment, blinding of participants, blinding of outcome assessors, incomplete outcome data, selective reporting, and other potential bias; it was evaluated as low, unclear, or high [17]. The reviewers divided the strength of evidence into high quality, medium quality, low quality, or very low quality evidence. The quality of evidence for each outcome was assessed using the grading of recommendations assessment, development and evaluation (GRADE) guideline development tool [18]. If two independent reviewers can not reach consensus, the final decision is made by a senior reviewer (He Liu).

### Statistical analysis

Meta-analysis was performed using Review Manager 5.4 (Cochrane Collaboration). Data used mean differences (MD) and odds ratio (OR), presented as 95% confidence intervals (CI). When *P* < 0.05 and 95% CIs excluding 1 for OR and 0 for MD, statistically significant differences were considered. Analysis of heterogeneity was carried out using the Chi^2^ test, and heterogeneity was evaluated using *I*^2^. When *I*^2^ values were < 25%, 25% to 50% and > 50%, the heterogeneity levels were correspondingly determined as low, medium or high. To explore the source of heterogeneity, sensitivity analysis or subgroup analysis should be performed if there is significant heterogeneity. The present study used a random effects model to combine the data, given the heterogeneity that can be expected.

We used the raw data from the selected literature as the primary source for extraction. When data were not presented in the original literature, we contacted the author to obtain the required information. Last resort, when means and SDs values were not available (the time first to request rescue analgesia [19–22], number of PCIA presses [19, 21, 22]), these values were imputed using the calculation methods of two statistical experts McGrath [14] and Cai [15]. Data from trial [22] with two intervention groups receiving different doses of DEX were combined into a single group as per the Cochrane Handbook [16].

### Assessment of publication bias

Since only 8 studies were included in this meta-analysis, the risk of publication bias was not assessed by examining the asymmetry of the funnel chart.

## Results

### Study selection

**Fig. 1:**
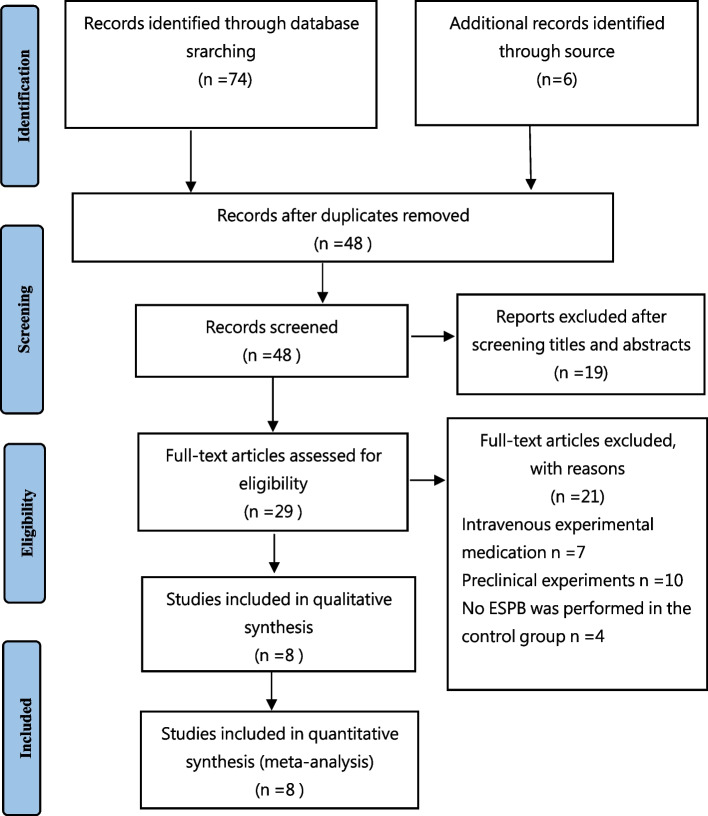
Flow diagram of the included and excluded studies

The search process was shown in Fig. 1. The literature search yielded a total of 80 articles from all databases. Of these, 32 articles were duplicated searches, so the process of study selection was performed on the remaining 48 articles. Sequentially, 19 articles were considered irrelevant studies after screening their titles and abstracts. The authors reviewed the full text of the remaining 29 relevant studies and excluded 21 studies from the final analysis because 7 studies were intravenous DEX, 10 studies were preclinical experiments and 4 studies were no ESPB was performed in the control group. Finally, 8 RCTs were included in the study [19–26]. Figure 1 represents the preferred reporting items for systematic reviews and meta-analysis (PRISMA) flow diagram, and summarizes the reasons for exclusion of records [13].

### Characteristics of included studies

The details of the included RCTs are summarized in Table 1. Of the 8 studies, 7 studies were from China [19, 21–26], 1 studies were from Egypt [20]. The level of nerve block localization was T 5 in 5 trials [19–21, 23, 26], T 4 + T 6 in 1 trial [22], T 3 in 1 trial [24], L 3 in 1 trial [25]. With the exception of 1 trial that used bupivacaine [20], 7 trials used another long-acting LAs ropivacaine [19, 21–26]. The DEX dosage was used in combination with LAs was 0.5 μg/kg in 3 studies [20, 22, 26], and 1 μg/kg in 5 studies [19, 21, 23–25]. The types of surgery are open thoracotomy [20, 26], video-assisted thoracoscopic lobectomy [19, 21–23], modified radical mastectomy [24], and posterior lumbar spine surgery [25].

**Table 1:** Characteristics of included randomized controlled trials

Study	Country	Surgery	Sample size(n)	Groups(n)	Dose of LAs	DEX dose	Block localization	Primary outcome
Elshal, 2021 [20]	Egypt	Thoracotomy	42	1.Bupivacaine + NS (21) 2.Bupivacaine + DEX(21)	0.25% 28 mL	0.5 μg/kg	Ultrasound (T5 spinous level)	The time first to request rescue analgesia
Gao X, 2019 [22]	China	Video-assisted thoracic surgery	108	1.Ropivacaine + 0.1 mg/kg dexamethasone(36) 2.Ropivacaine + 0.5 µg/kg DEX + 0.1 mg/kg dexamethasone(36) 3.Ropivacaine + 1 µg/kg DEX + 0.1 mg/kg dexamethasone(36)	0.375% 30 mL	0.5 μg/kg, 1 μg/kg	Ultrasound (T4 spinous + T6 transverse level)	VAS both at rest and with coughing during the 12 h after surgery
Gao Z, 2019 [19]	China	Video-assisted thoracoscopic lobectomy	90	1.Ropivacaine + NS (30) 2.Ropivacaine + 10 mg dexamethasone(30) 3.Ropivacaine + 1 μg/kg DEX (30)	0.5% 30 mL	1 μg/kg	Ultrasound (T5 spinous level)	Postoperative PCA use during the first 72 h
Rao, 2021 [21]	China	Video-assisted thoracoscopic lobectomy	95	1.Ropivacaine + NS (34) 2.Ropivacaine + DEX(34) 3.Ropivacaine + Nalbuphine (34)	0.5% 30 mL	1 μg/kg	Ultrasound (T5 spinous level)	PCA use during the first 72 h postoperatively
Wang Q, 2022 [26]	China	Open thoracotomy	60	1.Ropivacaine + NS (30) 2.Ropivacaine + DEX(30)	0.5% 30 mL	0.5 μg/kg	Ultrasound (T5 spinous level)	Duration of analgesia
Wang X, 2021 [24]	China	Modified radical mastectomy	60	1.Ropivacaine + NS (30) 2.Ropivacaine + DEX(30)	0.33% 30 mL	1 μg/kg	Ultrasound (T3 vertebral level)	Dosage of flurbiprofen at 48 h after surgery
Wang YH, 2022 [25]	China	Posterior lumbar spine surgery	120	1.Ropivacaine + NS (60) 2.Ropivacaine + DEX(60)	0.375% 20 mL	1 μg/kg	Ultrasound (L3 vertebral level)	VAS pain scores at rest and movement state after surgery
Yang, 2022 [23]	China	Thoracoscopic lobectomy	90	1.Ropivacaine + NS (28) 2.Ropivacaine + dexamethasone(27) 3.Ropivacaine + DEX (29)	0.5% 35 mL	1 μg/kg	Ultrasound (T5 and T6 spinous level)	The time to first postoperative remedial analgesia

*DEX* Dexmedetomidine, *NS* Normal saline, *Las* Local anesthetics, *VAS* Visual analogue score

### Risk of bias

**Fig. 2:**
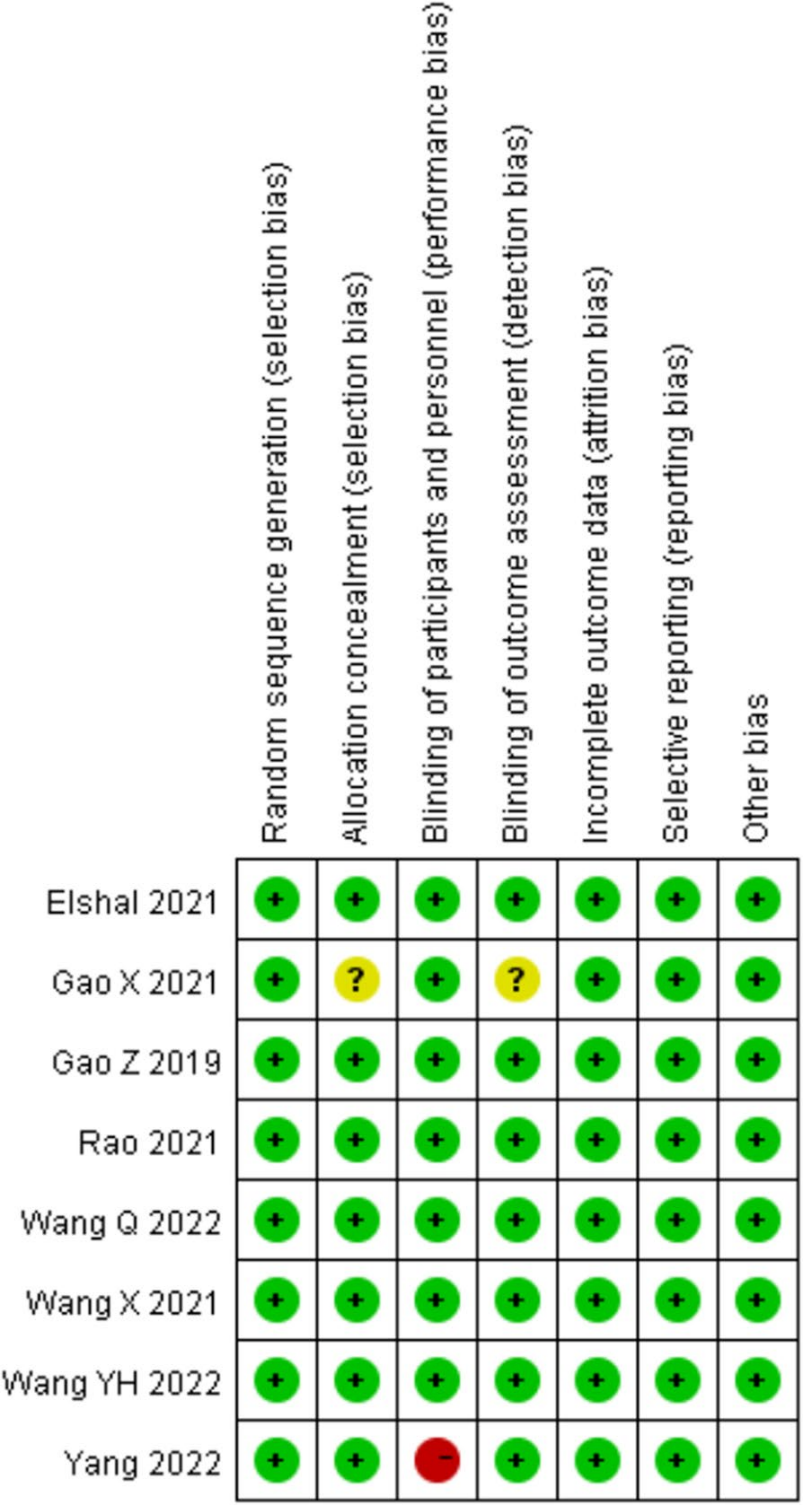
Cochrane Collaboration risk of bias summary: evaluation of bias risk items for each included study. Green circle denotes low risk of bias; yellow circle denotes unclear risk of bias; red circle denotes high risk of bias

A low level of overall risk of bias for included 8 trials. All patients were randomized to each group by appropriate methods, and allocation concealment was adequate in most studies. 1 of the studies reviewed lacked sufficient details in allocation concealment, and blinding of outcome assessors, in such case, we were conservative in our risk of bias evaluation by tending to classify trials as having an “unclear risk of bias” [22]. Furthermore, when the “attending anesthesiologist was informed about the grouping of the patients”, we judged the study to be “high risk of bias” [23]. A full risk-of-bias summary for all included studies is shown in Fig. 2.

### Grade

The factors affecting the quality of outcome include bias risk, inconsistency, indirection, imprecision and publication bias. In present study, the sample size of included studies was relatively small and the publication bias evaluation was not performed. The quality rating of the outcomes was reduced because of the unknown risk of publication bias and high heterogeneity. Main results are shown in Table 2.

**Table 2:** GRADE summary of findings

Outcomes	Number of patients (studies)	Effect		Certainty	Explanations
		Relative(95% CI)	Absolute(95% CI)		
The time first to request rescue analgesia	330 (5 RCTs)	-	MD 8.4 higher(4.7 higher to 12.1 higher)	⨁⨁◯◯Low	High statistical and clinical heterogeneity; unknown risk of publication bias
The rate of rescue analgesia	275 (4RCTs)	OR 0.33(0.17 to 0.65)	218 fewer per 1,000(from 295 to 97 fewer)	⨁⨁⨁◯ Moderate	Unknown risk of publication bias
The number of PCIA presses	233 (3RCTs)	-	MD 5.79 lower(7.24 lower to 4.35 lower)	⨁⨁◯◯ Low	High statistical and clinical heterogeneity; unknown risk of publication bias
The rate of PONV	450 (7RCTs)	OR 0.57(0.36 to 0.91)	96 fewer per 1,000(from 153 to 18 fewer)	⨁⨁⨁◯ Moderate	Unknown risk of publication bias
The incidence of hypotension	283 (4RCTs)	OR 1.01(0.37 to 2.74)	1 more per 1,000(from 40 fewer to 94 more)	⨁⨁⨁◯ Moderate	Unknown risk of publication bias
The rate of arrhythmia	283 (4RCTs	OR 0.76(0.19 to 3.07)	26 fewer per 1,000(from 95 fewer to 176 more)	⨁⨁◯◯ Low	High statistical and clinical heterogeneity; unknown risk of publication bias

*CI* Confidence interval, *MD* Mean difference, *OR* Odds ratio

GRADE Working Group grades of evidence; The quality considers: (1) within study risk of bias (methodological quality); (2) the directness of the evidence; (3) heterogeneity of the data; (4) precision of effect estimates; (5) risk of publication bias

High certainty: we are very confident that the true effect lies close to that of the estimate of the effect

Moderate certainty: we are moderately confident in the effect estimate; the true effect is likely to be close to the estimate of the effect, but there is a possibility that it is substantially different

Low certainty: our confidence in the effect estimate is limited: the true effect may be substantially different from the estimate of the effect

Very low certainty: we have very little confidence in the effect estimate; the true effect is likely to be substantially different from the estimate of effect

### Primary outcome

#### Time of the first request for rescue analgesia

**Fig. 3:**
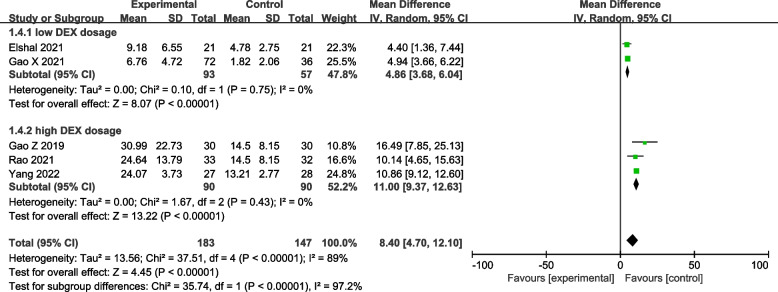
Forest plot of comparison: with VS. without DEX to the LAs in ESPB, time of the first request for rescue analgesia. IV, inverse variance

Five RCTs reported the time of the first request for rescue analgesia. A significant difference was found between the two groups (five studies [19–23]: MD = 8.4 h; 95% CI = 4.70 to 12.10; *p* < 0.00001; *I*^2^ = 89%; random-effects model; GRADE = Low; Fig. 3). Patients who used LAs mixed with DEX in ESPB had a significant delay in rescue analgesia.

Subgroup analyses were performed according to the dosage of DEX because the heterogeneity was high. Both the 0.5 μg/kg [20, 22] (MD = 4.86 h; 95% CI = 3.68 to 6.04; *p* < 0.00001; *I*^2^ = 0%; Fig. 3) and 1 μg/kg [19, 21, 23] (MD = 11.00 h; 95% CI = 9.37 to 12.63; *p* < 0.00001; *I*^2^ = 0%; Fig. 3) subgroups prolonged the time of the first rescue analgesia, and the prolonged time was more significant in the high dose subgroup. The data heterogeneity within the subgroup is low.

### Secondary outcome

#### Number of PCIA presses

**Fig. 4:**
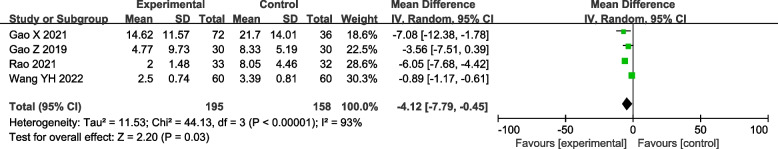
Forest plot of comparison: with VS. without DEX to the LAs in ESPB, number of PCIA presses. IV, inverse variance

Four RCTs reported the number of PCIA presses within 72 h after the operation. The pooled analysis showed that DEX as adjuvants significantly reduced the number of PCIA presses (four studies [19, 21, 22, 25]: MD = -4.12; 95% CI = -7.79 to -0.45; *p* = 0.03; *I*^2^ = 93%; random-effects model; GRADE = Low; Fig. 4).

**Fig. 5:**
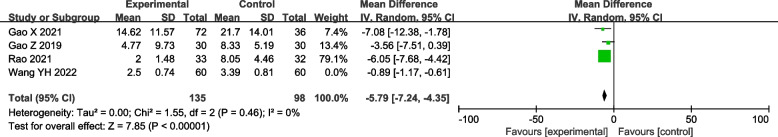
Forest plot of comparison: with VS. without DEX to the LAs in ESPB, number of PCIA presses, carried out sensitivity analysis and removed Wang YH’s research. IV, inverse variance

A sensitivity analysis was conducted to examine the source of heterogeneity. The pooled analysis results remained unchanged after excluding the data of Wang YH et al. [25], but the heterogeneity was low (MD = -5.79; 95% CI = -7.24 to -4.35; *p* < 0.00001; *I*^2^ = 0%; random-effects model; Fig. 5), indicating that this study is the main source of heterogeneity.

#### Number of remedial analgesia events

**Fig. 6:**
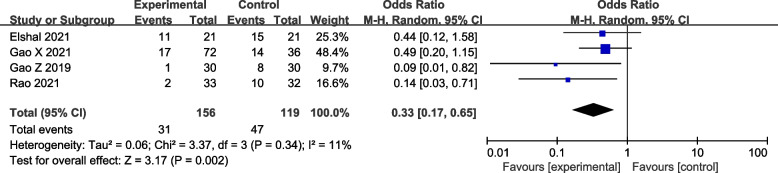
Forest plot of comparison: with VS. without DEX to the LAs in ESPB, number of remedial analgesia events. M-H, methods of merging dichotomous variables

Four RCTs reported the number of remedial analgesia events in the post-operative 72 h. Pooled analysis showed that the number of remedial analgesia events was significantly less in the DEX group (four studies [19–22]: OR = 0.33; 95% CI = 0.17 to 0.65; *p* = 0.002; *I*^2^ = 11%; random-effects model; GRADE = Moderate; Fig. 6).

#### Incidence of PONV

**Fig. 7:**
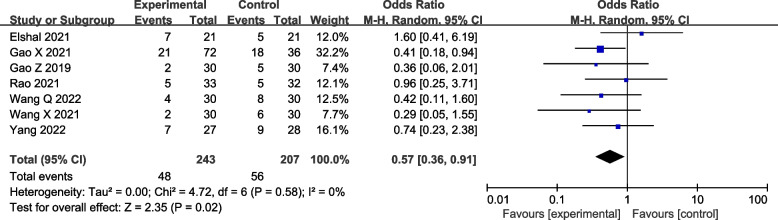
Forest plot of comparison: with VS. without DEX to the LAs in ESPB, incidence of PONV. M-H, methods of merging dichotomous variables

The incidence of PONV was reported in seven studies. The result indicated that DEX as an adjuvant decreased the incidence of PONV significantly (seven studies [19–24, 26]: OR = 0.57; 95% CI = 0.36 to 0.91; *p* = 0.02; *I*^2^ = 0%; random-effects model; GRADE = Moderate; Fig. 7).

#### Rate of hypotension

**Fig. 8:**
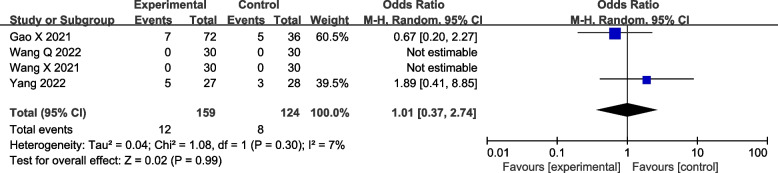
Forest plot of comparison: with VS. without DEX to the LAs in ESPB, rate of hypotension. M-H, methods of merging dichotomous variables

Four RCTs reported the rate of hypotension after surgery. The difference was not found to be significant (four studies [22–24, 26]: OR = 1.01; 95% CI = 0.37 to 2.74; *p* = 0.99; *I*^2^ = 7%; random-effects model; GRADE = Moderate; Fig. 8). In order to merge the research data to be more complete, although there were no hypotension events in the two studies, they were still included in the pooled analysis as the total number of events [24, 26].

#### Occurrence of arrhythmia

**Fig. 9:**
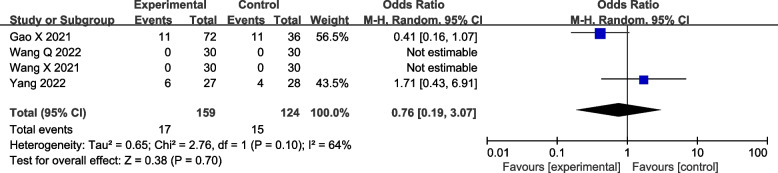
Forest plot of comparison: with VS. without DEX to the LAs in ESPB, occurrence of arrhythmia. M-H, methods of merging dichotomous variables

Arrhythmia is a common side effects after surgery. The arrhythmias in this study include sinus bradycardia and sinus tachycardia. The pooled analysis demonstrated that the occurrence of arrhythmia was no statistically significant difference between the two groups (four studies [22–24, 26]: OR = 0.76; 95% CI = 0.19 to 3.07; *p* = 0.70; *I*^2^ = 64%; random-effects model; GRADE = Low; Fig. 9). This result is moderately heterogeneous, but due to the inclusion of fewer studies and events, it is unable to carry out subgroup analysis, so the random effect model is used for analysis. The number of arrhythmia events in two studies was 0 and was still included in the pooled analysis to ensure the integrity of the data [24, 26].

## Discussion

More and more studies are on the application of DEX as an adjuvant to enhance the effect of nerve block. To our knowledge, this is the first meta-analysis to investigate the effect and safety of DEX combined with LAs for ESPB. Our systematic review and meta-analysis demonstrates that using DEX as adjunct in ESPB is associated with important positive effects in postoperative analgesia and reducing PONV. However, this method did not increase the risk of arrhythmia and hypotension.

We have three pooled analysis results (time of the first request for rescue analgesia, number of PCIA presses, and arrhythmia) with moderate or high heterogeneity. To explore causes of heterogeneity, we identified the clinical characteristics of individual RCTs, and possible reasons included: localization level of ESPB, LAs dose, DEX dose, types of surgery, and race. We conducted a subgroup analysis and found that different doses of DEX were the reason for the high heterogeneity of the time of the first request for rescue analgesia. Moreover the time of the high dose subgroup (1 μg/kg) was 11.00 h longer than that of the control group, while the low dose subgroup (0.5 μg/kg) was 4.86 h. Therefore, we have reasons to believe that a better analgesic effect can achieve by using a dose of 1 μg/kg in clinical application. When performing sensitivity analysis on the number of PCIA presses, we found that when removed the lumbar surgery, and only retained the thoracic surgery, the heterogeneity decreased significantly. The result shows that the type of operation is the main source of heterogeneity in the number of PCIA presses. Look forward to more RCTs on the application of ESPB in postoperative analgesia after lumbar surgery. This way, meta-analysis can be made using data from lumbar surgery alone. Because of the limited number of studies and events included in the rate of arrhythmia, subgroup and sensitivity analysis did not perform but use the random effect model for analysis.

Reduce the demand for analgesic drugs after the operation is an essential index of effectiveness. Reducing the demand for perioperative opioids is one of the goals of the current rapid rehabilitation program. Two RCTs in our review showed that the postoperative consumption of sufentanil in the control group was significantly higher than that in DEX group [23, 26]. This may be the reason for the significant decrease in the incidence of PONV in our pooled analysis. Previous meta-analysis has shown that DEX can prolong motor and sensory block time of nerve block, and our study has the same findings [19, 27].

Current perineural applications for DEX have relied on off-label uses of the drug. Therefore we must pay attention to drug safety. And the complications that often lead to adverse consequences were arrhythmia and hypotension. The results of some previous meta-analyses showed that DEX increased the odds of bradycardia and hypotension when used in brachial plexus block as an adjuvant for LAs [27–29]. Although, these side-effects were transient, reversible, did not require any intervention, and did not cause any long-term consequences in any of the patients. However, our results found no difference in the number of events between the DEX group and the control group. Dai et al. had come up with the same results as ours [30]. The possible reason is that the distribution of blood vessels in the cervical brachial plexus is more abundant than that between the muscle fascia of the erector spinae muscle, and the DEX absorb into the blood is faster and more. We noticed that ropivacaine was used in the data included in our study and Dai et al. [30]. Meanwhile, other studies have involved bupivacaine [27–29]. Ropivacaine may be more stable in hemodynamics than bupivacaine in clinical use. Similar views have reached from the results of other clinical studies [31, 32]. The above point of view recommended a new meta-analysis study to confirm.

Our meta-analysis has positive safety implications. The present review incorporates data from 303 patients who received DEX for the ESPB, and none reported any neurotoxicity symptoms or other neurologic sequelae. There is much evidence from in vitro and animal studies that applying DEX around the nerve may have a protective effect on LAs-induced inflammation [33, 34].

There are some limitations to our review. First, the high level of heterogeneity present across the pooled clinical outcomes. However, heterogeneity was successfully resolved with subgroup analysis and sensitivity analysis. Second, the number of studies included is relatively small, and the sample size was 10–50 patients per group, which may increased the possibility of class I errors. Since only eight articles were included in our study, we did not examine the asymmetry of the funnel chart to test the risk of publication bias. Third, the number of studies included in this analysis was not particularly great, and one of them was from Egypt [20], while the remaining seven studies were from China [19, 21–26], which may lead to the risk of bias. Nevertheless, there are 10 pre-registered clinical trials from different countries underway, and we will continue to pay attention to the relevant research results in the future and go on to update the current meta-analysis. Fourth, the final number of included papers shows that Chinese research is active in this field, but no Chinese databases were included in the pre-planned search database.

In contrast, our review has several points of strength. The kinds of literature included were all RCTs. All the studies are completed with high quality and have good contrast. We have found the sources and reasons for some of the high heterogeneity of the pooled results. In the course of subgroup analysis, while used a variety of doses of DEX, we detected a dose–response. Evidence suggests that DEX produces a dose-dependent prolongation in postoperative analgesia duration. These factors underscore the validity of our findings.

## Conclusion

Overall, the results of our systematic review and meta-analysis suggest that DEX as an adjuvant to LAs in ESPB, particularly at doses of 1 μg/kg, holds great potential for clinicians wishing to prolong the duration of anesthesia and reduce the demand for analgesics after surgery. In addition, the combination does not increase the incidence of arrhythmia and hypotension. Future research should focus on whether this combination will prolong motor block duration, resulting in the risk of delayed recovery. At the same time, we encourage more relevant research to update this meta-analysis.

## Supplementary Information

**Additional file 1.**

## Data Availability

The datasets used and/or analysed during the current study are available from the corresponding author on reasonable request.

## Abbreviations

DEX: Dexmedetomidine
LAs: Local anesthetics
ESPB: Erector spinae plane block
PCIA: Patient controlled intravenous analgesia
PONV: Postoperative nausea and vomiting
MD: Mean difference
CI: Confidence interval
RCT: Randomised controlled trial
OR: Odds ratio
FDA: Food and Drug Administration
GRADE: Grading of recommendations assessment, development and evaluation
