# Supplementary material for: The effect and safety of dexmedetomidine as an adjuvant to local anesthetics in erector spinae plane block: a systematic review and meta-analysis of randomized controlled trials
Source: BMC Anesthesiol. 2023 Feb 27;23:61. doi: 10.1186/s12871-023-02019-x (PMC9969627; doi:10.1186/s12871-023-02019-x)
Supplement: Supplementary file 1 — Additional file 1. [file 12871_2023_2019_MOESM1_ESM.doc]

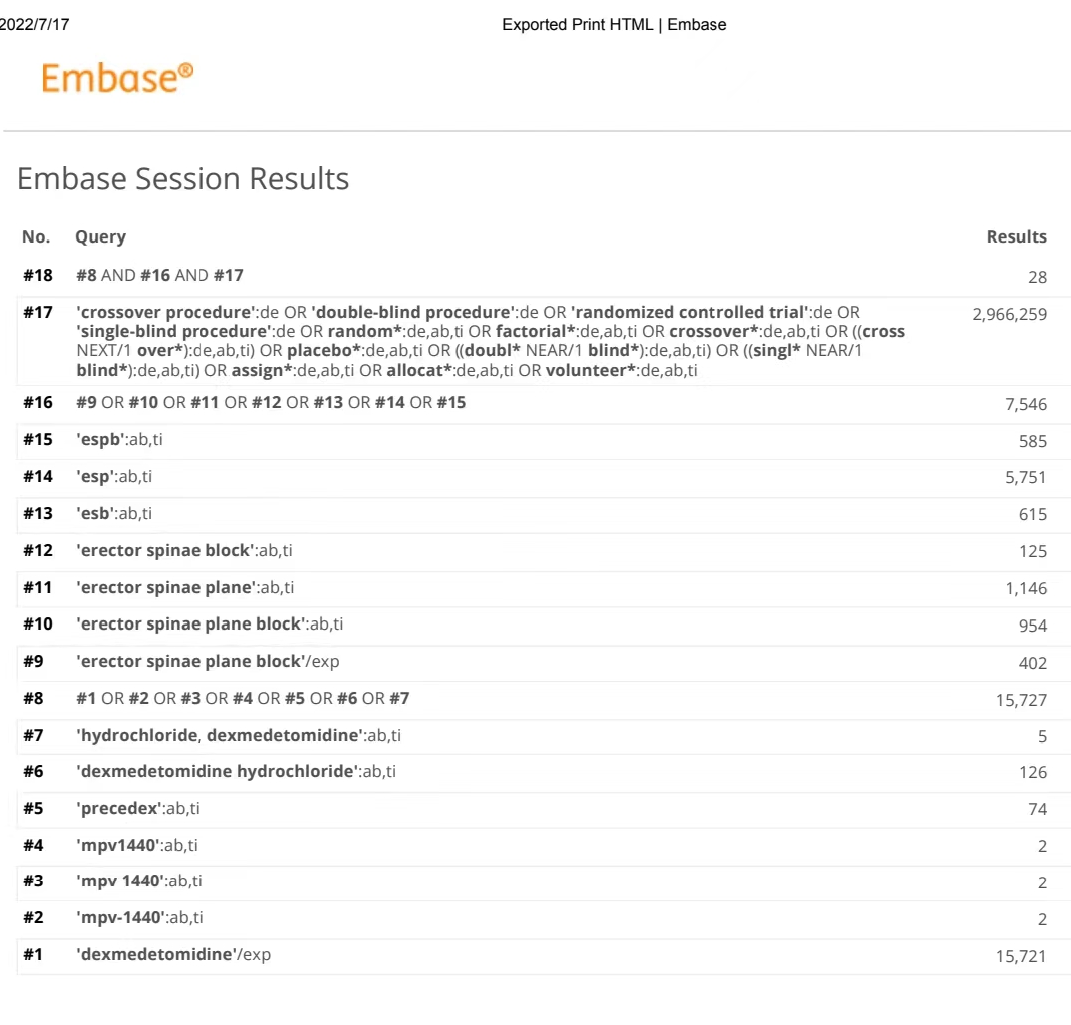


Cochrane Central Register of Controlled Trials

#1 (erector spinae):ti,ab,kw.

#2 (erector spinae plane):ti,ab,kw.

#3 MeSH descriptor: [Paraspinal Muscles] explode all trees.

#4 #1OR#2OR#3

#5 (Nerve Block):ti,ab,kw

#6 MeSH descriptor: [Nerve Block] explode all trees

#7 MeSH descriptor: [Anesthesia, Local] explode all trees

#8 #5OR#6OR#7

#9 ("dexmedetomidine"):ti,ab,kw

#10 #4AND#6AND#9

#10 with Publication Year from 1900 to July 2022.

Web of Science

1: (((((AB=(erector spinae plane)) OR AB=(erector spinae plane block)) OR AB=(erector spinae block)) OR AB=(ESB)) OR AB=(ESP)) OR AB=(ESPB)

2: ((((((AB=(erector spinae plane)) OR AB=(erector spinae plane block)) OR AB=(erector spinae block)) OR AB=(ESB)) OR AB=(ESP)) OR AB=(ESPB)) AND AB=(Dexmedetomidine)

PubMed Search Strategy

(((((((erector spinae plane[Title/Abstract]) OR (erector spinae plane block[Title/Abstract])) OR (erector spinae block[Title/Abstract])) OR (ESB[Title/Abstract])) OR (ESP[Title/Abstract])) OR (ESPB[Title/Abstract])) AND (((((((Dexmedetomidine[Title/Abstract]) OR (MPV1440[Title/Abstract])) OR (MPV 1440[Title/Abstract])) OR (MPV1440[Title/Abstract])) OR (Precedex[Title/Abstract])) OR (Dexmedetomidine Hydrochloride[Title/Abstract])) OR (Hydrochloride, Dexmedetomidine[Title/Abstract]))) AND ((randomized controlled trial[pt] OR controlled clinical trial[pt] OR randomized[tiab] OR placebo[tiab] OR drug therapy[sh] OR randomly[tiab] OR trial[tiab] OR groups[tiab] NOT (animals [mh] NOT humans [mh]))) Sort by: Most Recent
